# Beyond Kuhnian paradigms: Normal science and theory dependence in ecology

**DOI:** 10.1002/ece3.10255

**Published:** 2023-07-04

**Authors:** Craig A. Layman, Andrew L. Rypel

**Affiliations:** ^1^ Center for Energy, Environment, and Sustainability Wake Forest University Winston‐Salem North Carolina USA; ^2^ Department of Wildlife, Fish & Conservation Biology, and Center for Watershed Sciences University of California, Davis Davis California USA

**Keywords:** biodiversity loss, food webs, paradigm, philosophy of science, scientific method, scientific revolutions, theory dependence, Thomas Kuhn

## Abstract

*The Structure of Scientific Revolutions* by Thomas Kuhn has influenced scientists for decades. It focuses on a progression of science involving periodic, fundamental shifts—revolutions—from one existing paradigm to another. Embedded in this theory is the concept of normal science, that is, scientists work within the confines of established theory, a process often compared to a type of puzzle‐solving. This Kuhnian aspect of scientific research has received little attention relative to the much‐scrutinized concepts of revolutions and paradigms. We use Kuhn's normal science framework to reflect on the way ecologists practice science. This involves a discussion of how theory dependence influences each step of the scientific method, specifically, how past experiences and existing research frameworks guide the way ecologists acquire knowledge. We illustrate these concepts with ecological examples, including food web structure and the biodiversity crisis, emphasizing that the way one views the world influences how that person engages in scientific research. We conclude with a discussion of how Kuhnian ideas inform ecological research at practical levels, such as influences on grant funding allocation, and we make a renewed call for the inclusion of philosophical foundations of ecological principles in pedagogy. By studying the processes and traditions of how science is carried out, ecologists can better direct scientific insight to address the world's most pressing environmental problems.

## Graphical abstract

We use Thomas Kuhn's “normal science” framework to reflect on the way ecologists practice science. This involves a discussion of how theory dependence influences each step of the scientific method, and how past experiences and existing research frameworks guide the way ecologists acquire knowledge. We illustrate these concepts with ecological examples, including perspectives on food web structure and the biodiversity crisis, emphasizing that the way one views the world influences how that person engages in scientific research.

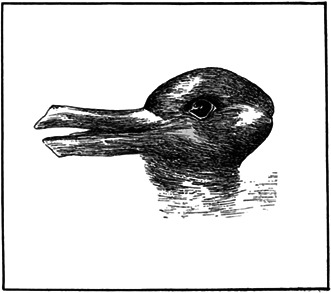

## 1 INTRODUCTION

Understanding the genesis and architecture of scientific inquiry is critical for intellectual progress and the study of processes and traditions identifies pathways for directing scientific insight toward desired goals. Ecologists, like practitioners in other scientific fields, consider how historical and societal contexts influence scientific practice. *The Structure of Scientific Revolutions* (Kuhn, 1962), one of the most influential books of the 20th century, challenged existing views regarding the frameworks of scientific inquiry. Philosophers and historians have extensively studied the applicability and implications of Thomas Kuhn's ideas and the book has inspired scientists, including ecologists, to reexamine their fields' histories, methodologies, and biases. Ecology is defined as the study of patterns and processes in natural systems, and the philosophy of ecology involves fundamental principles that define the field as well as the methods used to investigate these principles (Keller & Golley, 2000). The philosophy of ecology has direct implications for applied questions, such as how natural resources are managed and conservation policies are developed. Revisiting Kuhn's ideas about the progress of science is a logical starting point.

A common philosophical pursuit for ecologists is exploring the role of “paradigms” for the field and its subdisciplines (e.g., Rypel et al., 2021; Sheehan, 2019; Wu & Loucks, 1995). Kuhn articulated a theory of scientific progress that was different from the prevailing notion of a cumulative, linear process of the accumulation of scientific knowledge. He argued that science advances through major revolutions, in which one prevailing scientific paradigm is completely replaced by another. Ecologists have long debated if there are true Kuhnian paradigms in the field, and how these paradigms (and revolutions) frame scientific progress (e.g., Austin, 1999, Hengeveld & Walter, 1999, Simberloff, 1980, Walter & Hengeveld, 2000, and see *Ecology* v. 83, no. 6). There are diverse perspectives regarding the applicability of paradigms and revolutions to ecology. Some lean toward acceptance of the Kuhnian model and seek to identify which ecological frameworks best qualify according to Kuhn's paradigm criteria. Others reject Kuhnian ideas because characteristics of ecology render it unique compared to the physical sciences on which Kuhn based many of the details in *The Structure of Scientific Revolutions* (1962, hereafter, *Structure*. Any subsequent references to Kuhn allude to this text unless noted).

Referring to Cuddington and Beisner (2005), we agree with Robert Paine in the Foreword that “Whether our discipline has paradigms or not seems immaterial” and the book editors in the Conclusion: “The answer to the question of what ecologists mean when they use the phrase paradigm shift is likely to be: anything.” Yet, the ecological literature drawing on Kuhn revolves around four semantic questions. *Does ecology have paradigms? If so, what are the guiding paradigms? What is a scientific revolution? Have there been Kuhnian revolutions in ecology?* In addition to the extensive literature on these questions, we have seen this semantical push in workshops, seminars, and classes where Kuhnian ideas are broached. Even with focused efforts to direct the conversation to other aspects of *Structure*, discussions inevitably shift back to paradigms and revolutions, rendering a survey of Kuhnian ideas incomplete. Consistent with this observation, even though this paper has different objectives, it is hard to avoid some discussion of paradigms. Yet, there is more in *Structure* that can inform our pursuit of fundamental ecological principles and how we apply them to address environmental problems.

To this end, we focus on two concepts central to *Structure*: normal science and theory dependence. Kuhn devoted much text in *Structure* to the practice of normal science—the activities that occur between revolutions, the primary day‐to‐day doings of scientists. We highlight the fundamental characteristics of normal science as Kuhn described it and emphasize how it is essential to ensuring scientific progress. Tacit in normal science is the concept of theory dependence—the way we see the world is the way we are taught to see the world. We explore how this concept affects each step of the scientific method and use ecological case studies to illustrate it. We conclude with a discussion of how Kuhnian ideas inform ecological research at practical levels, such as influences on grant funding allocation, and we emphasize the need for the inclusion of philosophical principles in teaching and mentoring programs.

We extend the ideas and perspectives of Kareiva et al. (2018) by linking to philosophical foundations developed by Kuhn; this provides an additional framework to explore the various case studies included in the Kareiva et al. (2018) text. Our essay is not a philosophical argument of whether Kuhn's view of scientific progress is correct and it does not address what an ecological paradigm is. There may be intrinsic flaws to the Kuhnian view of science (e.g., Bird, 2002; Fodor, 1984; Fuller, 2004; Mladenović, 2022; Sanbonmatsu & Sanbonmatsu, 2017) and we do not review these discussions. Instead, the focus is on less well‐studied Kuhnian ideas that relate to the philosophical underpinnings of ecology. Although such calls for philosophical thought in ecology are often found in the literature, our emphasis on normal science and theory dependence highlights different discussion foci.

## 2 NORMAL SCIENCE

Kuhn defined paradigm as “universally recognized scientific achievements that for a time provide model problems and solutions to a community of practitioners” (Kuhn, 1962). Paradigms are constructs that frame scientific endeavors, allowing scientists to define avenues of relevant inquiry and to identify what tools or methods should be used to address questions deemed important. Research within a prevailing paradigm is labeled normal science—research based on past scientific achievements that form the foundation for further research. Normal science is based on the assumption that scientists know what the world is like and that they already have an accurate depiction of the way nature behaves. Much of normal science is, therefore, devoted to actualizing the promise of this understanding by increasing the match of observations and experimental results to common knowledge. Kuhnian normal science is neither intended nor expected to produce novel discoveries or significant alterations to an existing paradigm. Scientists are expected to expand and contextualize existing scientific theories, not disprove them. Anomalous findings may discredit the scientist, not the accepted theory. Kuhn likened normal science to puzzle‐solving—successful scientists are those who are successful puzzle solvers. It is known a priori that puzzles have solutions, so scientists are not bound by the fear they may be working on an impossible problem.

Rigid and rigorous disciplinary training is core to normal science, from theoretical frameworks to the basic information in textbooks. Kuhn suggests students may lean toward the wisdom of accepted figures in the field at the expense of evaluating evidence for themselves. Indeed, there is evidence for these types of biases within the field (Leimu et al., 2008). Scientists do not learn in a vacuum. Pedagogical histories—and, more broadly, societal contexts—shape the way we view nature. Kuhn writes: “An apparently arbitrary element, compounded of personal and historic accident, is always a formative ingredient of the beliefs espoused by a given scientific community at a given time (p. 4).” He describes highly convergent sets of activities in normal science, all of which are directed toward common goals.

The three primary activities in normal science are the determination of significant facts, matching of facts with theory, and articulation of theory. Because the basic tools and assumptions in normal science are agreed upon, a scientist is free to explore the intricate details of a particular system, filling knowledge gaps. A researcher does not have to consistently recreate the field or develop new tools—they are already in place. By focusing on specified subsets of predefined problems, Kuhn suggests the depth of research possible in normal science is “unimaginable” and allows for solving problems that scientists otherwise “could scarcely have imagined and would never have undertaken (p. 25).”

An inclination is to view normal science as insignificant in the broader acquisition of scientific knowledge. After all, the book is titled *The Structure of Scientific Revolutions* not *The Structure of Normal Science*. A point to consider is how the use of the term “normal” may have influenced the entire discourse regarding normal versus revolutionary science. Many view the Kuhnian description of normal science as a criticism. They view their science as being important even if it does not meet the expansive definition of revolutionary that Kuhn outlines. Many believe Kuhn's message is that only revolutionary science matters, again, think of the title of the book and the seemingly countless pages that have been written about scientific revolutions. This was definitively not his view—he went to great lengths to discuss the critical and fundamental role of normal science in the scientific process.

Perhaps, if Kuhn simply used the word “science,” or a phrase such as “fundamental science,” it would have tempered resistance to his theory. Such semantical problems in ecology are pervasive. Peters (1991) pointed out that linguistic barriers hold back progress in the ecological sciences. For example, a term may be defined broadly so two people may not recognize they are talking about different concepts when referring to the same term. The same term may have varied meanings to scientists based on their a priori experience. The phrase normal science may be one such linguistic barrier. Moving beyond the semantics of normal science may allow for more nuanced perspectives on underlying concepts that transcend the phrase itself.

## 3 THEORY DEPENDENCE

A foundation of Kuhn's philosophy, especially regarding normal science, is that science is inherently theory‐dependent, that is, scientific endeavors are framed, explored, and assessed according to prevailing theory. Whereas the Kuhnian terms paradigm and revolution may be familiar to many ecologists, explicit exploration of the role of theory dependence is much less so. Theory dependence in scientific investigation is not a uniquely Kuhnian idea (Boyd, 1991; Feyerabend, 1965; Grandy, 1973; Hanson, 1958). The phrase is not explicitly called upon in *Structure*, but theory dependence largely frames the context. This concept builds on the concept of theory‐ladenness of observation as espoused by Hanson (1958)—what one sees depends on background context and the perceiver's beliefs and experiences.

**FIGURE 1:**
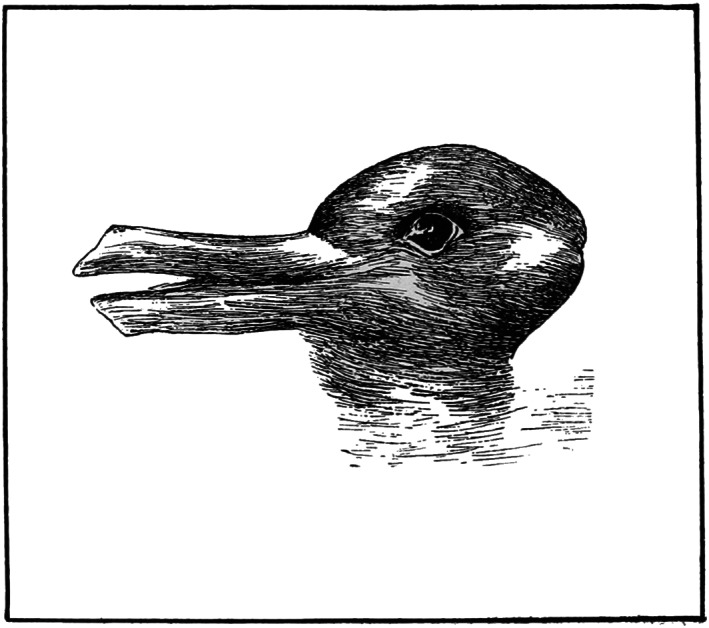
Optical illusion of a duck and rabbit from Popular Science Monthly, Volume 54 (1899). Drawn by Joseph Jastrow.

Kuhn rendered the ideas of Hanson regarding the theory‐ladenness of observations more encompassing, highlighting how theory dependence affects each stage of the scientific method. Theory dependence confines and directs the way the world is viewed, identifies the questions we ask, outlines the methodologies employed to investigate these questions, and influences how results are interpreted. Critical to the concept of theory dependence is that observation is not independent of one's previous experience. In Kuhn's words, “What a man sees depends both upon what he looks at and also upon what his previous visual‐conceptual experience has taught him to see (p. 133).” A classic (admittedly simple) example is the duck–rabbit figure (Figure 1), an ambiguous drawing that could look like either a duck or a rabbit depending on the observational perspective. A person that has no prior experience with a rabbit may see a duck, but one who does not know that ducks exist may see a rabbit.

Kuhn argues this phenomenon can be traced to the education of young scientists who are trained in prevailing dogma. Students are taught using examples that directly replicate or simulate classic experiments and observations within the current accepted state of the field. They are then challenged to investigate similar phenomena that relate to these ideas. Theory dependence forms a nexus with an interlocking set of commitments: conceptual, theoretical, instrumental, and methodological. In sum, theory dependence in science is “based firmly upon a settled consensus acquired from scientific education and reinforced by subsequent life in the profession” (Kuhn, 1959).

To illustrate with an ecological example, we compare two divergent views of food webs, that is, networks of consumer–resource interactions among a group of organisms, populations, or aggregate trophic units (Winemiller & Polis, 1996). A scientist trained in mathematics or statistics may focus on the word *network* in the definition (Dunne et al., 2002; Lau et al., 2017), viewing food webs through a quantitative lens. One from a more empirical background could see *consumer–resource interactions* as the core aspect of the definition (e.g., Polis, 1991). This simple difference in perspective could give rise to divergent modes of conceptualizing and studying food webs—and thus different ways to view the world. Take two well‐cited papers as examples: Williams and Martinez (2000) and Winemiller (1990). The former starts from the premise that quantitative models can be used to universally represent the structure of food webs; the latter starts with empirical data on consumer–resource relationships (diets) and works toward broader descriptors of food web structure. Note that they use the same tools: empirical data (drawn from the literature in the first study, the second study uses empirically compiled data) and modeling (the first study with modeling as the primary tool, the second study building model results from the empirically based data).

Despite the same tools, the different a priori perspectives (and the different workflow trajectories) yield rather different insights into the fundamental nature of food webs (Layman et al., 2015). Williams and Martinez (2000) find that the simple rules that define their model (e.g., randomly assigned feeding relationships) can predict the structure of actual food webs. Winemiller (1990) described an alternative world in which food webs are vastly more complex than suggested by modeling approaches. One approach yields a view that simple rules govern nature, whereas the other reveals that the sheer complexity of interactions overrides attempts at simplification. What each subdiscipline trained its researchers to see is what they saw—different world views even within the relatively narrow bounds of food web ecology. One sees general principles whereas another sees intricacies that belie generalization. One sees a duck, the other sees a rabbit.

## 4 THE SCIENTIFIC METHOD

**FIGURE 2:**
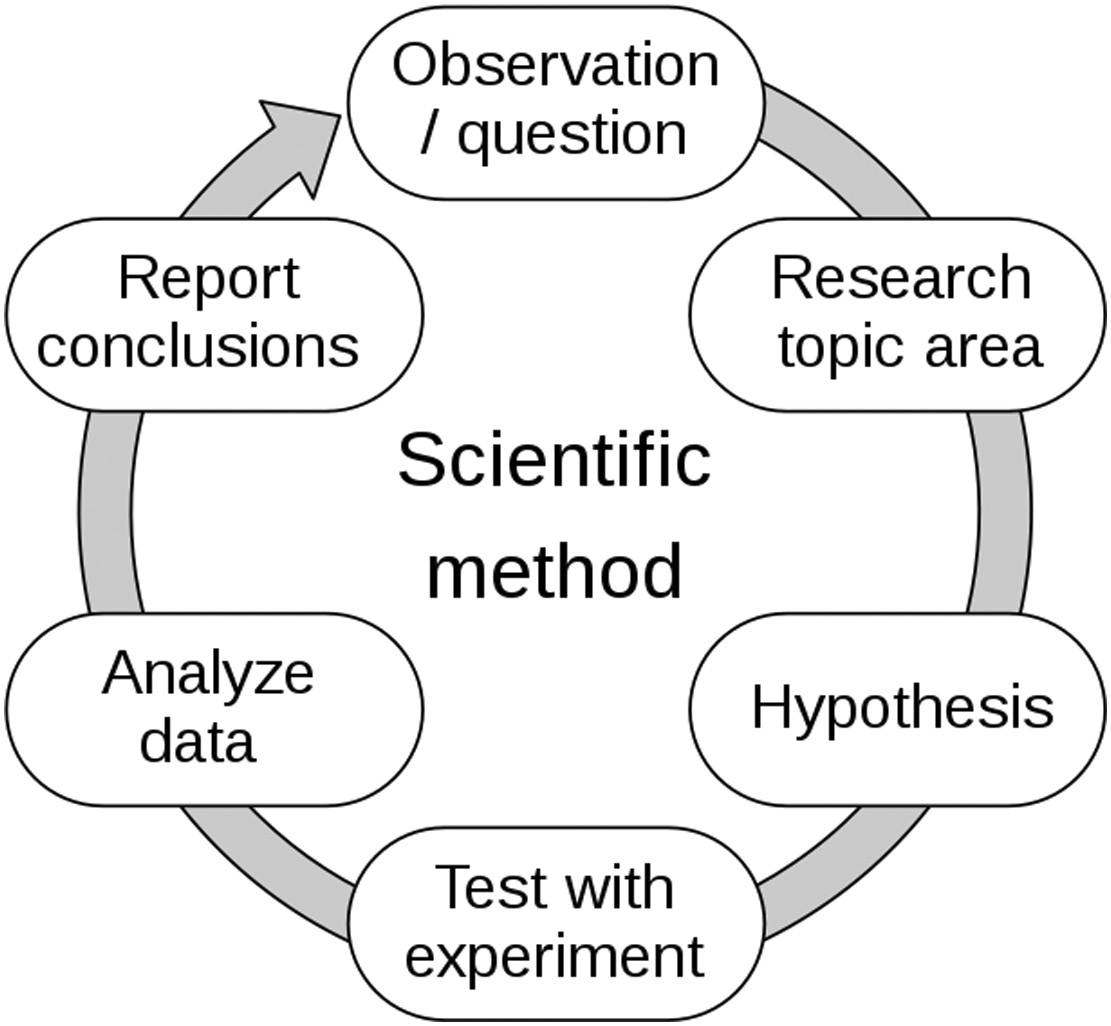
Standard depiction of the scientific method. This image was accessed from Wikimedia Commons.

Kuhn articulated how theory dependence can affect each stage of the scientific method (Figure 2). The observation stage is directly influenced by one's experience and training—observers provided with the same sets of visual (or other perceptual) data can draw strikingly different conclusions because of previous experience. In a basic example from *Structure*, when looking at a map, a cartographer sees landmarks in space, whereas a young child only sees lines on paper. It can be argued that classic debates in ecology can be understood by identifying the standpoint from which scientists viewed different phenomena. For example, the divergent views of Tansley, Gleason, and Clements regarding ecosystem structure and succession may simply have sprung from different ways of perceiving vegetation in ecosystems (Allen & Hoekstra, 1992). In Kuhn's words, each man saw what his prior experience and background taught him to see. Andersen et al. (2019) describe this concept more broadly, identifying categories of assumptions scientists make (based on their training and background experiences) that, in turn, influence observation and the scientific method: how the world is (ontology), what we can know about it (epistemology), or how science ought to be practiced (norms).

Hypotheses stem directly from observation and are likewise influenced by experience and training. Kuhn argues that most scientific research is focused on the elaboration of existing theories that are accepted as true, and thus hypothesis generation is unlikely to stray from accepted dogma and theoretical frameworks: “One of the things a scientific community acquires with a paradigm is a criterion for choosing problems that, while the paradigm is taken for granted, can be assumed to have solutions (p. 37).” Radical hypotheses in this framework are viewed less favorably than those that conform to or confirm tenets of prevailing theory.

The testing/experimentation stage should be the most objective stage of the scientific method but theory dependence is still influential. Experiments are designed in the context of the prevailing theory and thus hypotheses are generated from inherently theory‐dependent observations. Researchers tend to design confirmatory experiments, not those that challenge the premises of existing constructs (Brady, 1982; Graham & Dayton, 2002; Loehle, 1987). To this end, it is common for researchers to seek systems to test hypotheses where their hypotheses are most likely to hold and the likelihood of confirmation is increased by subjective site or organism selection.

Analyzing, evaluating, and reporting data of scientific results are analogous to the initial observation stage. That is, researchers have an idea of what form results should take and thus may be biased to accept results that align with prevailing theories (Loehle, 1987). Hall (2021) provides insight into this concept based on phenomena he labels “Failure of the Nerve” and “Failure of the Imagination.” The former suggests that a result may not be accepted, even if obvious, if it is “far enough out of common‐sense experience, outside the box or the Overton window, the mind balks (p. 87).” Hall also suggests a result could be misinterpreted because of the Failure of the Imagination, that is, a finding is not acknowledged because of the lack of tools or experience to properly envision that particular finding. As such, we are often poorly equipped and inexperienced in thinking about ecological surprises—unexpected findings about the environment (Lindenmayer et al., 2010). More fundamentally, Kareiva and Marvier (2018) point out that holding on to existing beliefs despite contradictory evidence is inherent to the human condition.

The effect of preconceived notions on the evaluation of scientific observations is illustrated by Chabris and Simons (2010) in *The Invisible Gorilla*. The book is titled after the famous psychological experiment in which people are tossing a basketball to one another. The audience is instructed to watch a video and count the number of times the ball is passed. In the middle of the video, a gorilla walks past the people passing the basketball. Remarkably, as many as half of the people (in numerous replicates of the experiment, including our own) do not report seeing the gorilla. Once told about the gorilla, its presence is obvious in a subsequent review of the video. Further, the audience is asked what letter is on the back wall of the room—few people identify this letter. The audience members are so attuned to the pass‐counting exercise that they do not see something that is otherwise apparent. They are told to watch the basketball and that is what they do—at the expense of observing other real aspects of that experimental world. If told to look for a gorilla, everyone watching the video would see a gorilla.

Theory‐dependent components of the scientific method are self‐reinforcing, feeding into existing theory. Confirmation bias is relevant here—a tendency to support one's theory or not to seek or use contradictory evidence (Loehle, 1987; Silliman & Weir, 2018). This leads to publication bias, that is, the tendency to publish positive results (e.g., those consistent with an existing theory or that support a hypothesis) instead of negative ones (Fanelli, 2010; Jennions & Moller, 2002; Lortie et al., 2007; Martinez‐Abrain, 2013; Wood, 2020). Publication bias further strengthens the role of theory dependence, as studies that are inconsistent with prevailing theory are left unpublished, which is labeled the file drawer effect (Csada et al., 1996; Dalton et al., 2012). Evidence could have accumulated that contradicted accepted wisdom, but it remained inaccessible to the scientific community. Such tendencies, associated with the practice of normal science, inform how ecologists acquire knowledge, how it is disseminated, and how it is applied to address real‐world problems.

## 5 THEORY DEPENDENCE BOTH DRIVES AND CONSTRAINS PROGRESS IN ECOLOGY

The field of salt marsh ecology was rooted in research from the 1950s and 1960s at the University of Georgia's Marine Institute on Sapelo Island, Georgia. A research symposium held at the Institute in 1958 generated early interest both in the ecology of salt marshes and, more broadly, how research in marshes could contribute to the emerging field of ecosystem ecology (Odum & Smalley, 1959; Wiegert & Evans, 1967). This led to major ecosystem ecology‐centric endeavors, such as the International Biological Program (Hagen, 1992). Important research directives were generated, for example, grazers and other top‐down forces were relatively unimportant in regulating marsh production and consumption of dead plant material (detritus) fueled marsh food webs (Marples, 1966; Smalley, 1960; Teal, 1962). These concepts became widely accepted truths and provided the foundation for the dominant bottom‐up, detrital‐based theory in the field. As Kuhn outlines for other fields, these ideas were entrenched in textbooks used to train students and other practitioners (e.g., Mitsch & Gosselink, 2000; Odum, 1953; Pomeroy & Wiegert, 1981). For nearly 50 years, this framework provided context for the majority of ecological investigations in marshes globally, as well as other marine macrophyte‐dominated systems, such as seagrasses and mangroves, to which this viewpoint was extrapolated (Odum & Heald, 1975; Ogden, 1980; Zieman & Zieman, 1989). This fueled remarkable advances in our understanding of salt marshes, and ecosystems more generally, intricately refining details on the mechanisms of physiochemical control of ecosystem processes (some of the myriad examples include Bradley & Morris, 1990, Currin et al., 1995, Howes et al., 1981, King et al., 1982, Mendelssohn et al., 1981).

Theory dependence is at the core of understanding critical conceptual shifts that occurred in the 2000s. Early marsh studies employed various techniques, many based on correlative patterns and direct observations, to examine the impacts of consumers. In short, they supported the concept that few animals ate live salt marsh grass. Scientists observed high densities of invertebrate grazers associated with areas of dying and senescent marsh grass and suggested animals were attracted to these areas because of the abundance of dead organic matter—they did not test alternative hypotheses that grazers could be causing the grass death. In a salt marsh caging experiment focused on a common detritivore, the periwinkle *Littoraria irrorata*, there was evidence that snails graze live grass and control *Spartina alterniflora* (saltmarsh cordgrass) biomass. But this observation was not considered further because of the dominance of prevailing bottom‐up theories (Stiven & Kuenzler, 1979). Ecologists were seeing the effects of grazing—but that was not what they expected to see. Later studies explored the grazing possibility further and showed top‐down effects of insects, nutria, geese, and horses (Smith & Odum, 1983; Taylor & Grace, 1995; Turner, 1987). Yet, these results were deemed as limited phenomena in idiosyncratic situations, and thus unimportant compared to overriding bottom‐up forces (e.g., Mitsch & Gosselink, 2000). The bottom‐up theory proved highly resistant to change despite evidence to the contrary.

The bottom‐up paradigm remained intact until the late 1990s. A paradigm shift started with some basic observations about *Littoraria* (Silliman & Zieman, 2001): They graze directly on live *Spartina* tissue and their densities are often high (up to 700/m^2^). Researchers experimentally manipulated *Littoraria* densities, testing if snails had a top‐down effect—a hypothesis many scoffed at. However, experiments showed that: (1) *Littoraria* harm live blades with their radulae, (2) they passively farm fungi on the marsh grass surface, and (3) these activities result in the suppression of marsh grass biomass (Silliman & Bortolus, 2003; Silliman & Zieman, 2001). These findings were not applied to explain similar patterns in other systems. For example, McKee et al. (2004) published a study soon after that was focused on widespread salt marsh die‐off and discounted that snails played any functional role, based on observations that they only seemed to be consuming dead grass. Others conducted experiments to disprove the strength of top‐down control (Kiehn & Morris, 2009), despite mounting evidence in support of it (Bertness & Silliman, 2008; Pennings & Silliman, 2005; Silliman et al., 2005; Silliman & Bertness, 2002; Silliman & Bortolus, 2003). Silliman often faced not‐in‐my‐backyard responses, that is, perhaps top‐down control may be important elsewhere but not in the system a person knows well (CAL, *personal observations*). Again, the decades‐long dominant theory that framed how ecologists studied salt marshes created challenges to view salt marsh dynamics in new ways. Most scientists were trained to see rabbits—but looking at the system differently revealed ducks.

This case study illustrates the two sides of normal science and theory dependence: they drive and constrain progress. The challenge in this context is to balance the benefits of doing normal science while allowing for intellectual freedom to explore new and risky ideas—Kuhn's “essential tension” (Kuhn, 1959). Discussion of this creativity versus constraint tension is an important component of pedagogy and the basic training of ecologists. Furthermore, it could be more explicitly addressed when ecologists are asked to deal with conservation challenges (Kareiva et al., 2018). We focus on one example but such instances of theory dependence constraining and driving progress in the field are commonplace. For example, consider the viewpoints that invasive species are necessarily “bad” (Schlaepfer, 2018) or that introducing wolves to Yellowstone National Park is implicitly “good” (Marris, 2018). In each case, studies contradicting these perspectives met resistance, yet the prevailing theories allowed research consistent with the constructs to flourish.

## 6 CHALLENGES TO THEORY

We use one of our own experiences with the biodiversity crisis as another example of research challenges associated with theory dependence. The classic prediction from Norman Myers (1979) that 50% of all species would be extinct by the year 2000 framed an entire research discipline focused on minimizing biodiversity losses and understanding the implications of these losses (Fahrig, 2003; Myers et al., 2000). Related to these efforts are estimates of species extinction rates, framed by the prevailing view that species extinctions are pervasive and greatly exceed historical rates. In the early 2000s, we were working with colleagues on a paper that suggested conservation biologists would be well served to shift away from making extinction rate estimates. We stated that biodiversity loss is occurring, is detrimental to the functioning of ecosystems, and efforts need to be made to stem these declines. But, already having the knowledge framework and tools to move forward with protecting ecosystems and constituent species, we do not need to make speculative estimates of species extinction rates. With no means to accurately verify rates with available data, taking this approach could undermine the public's willingness to accept ecological assessments, reduce trust in scientific institutions, and hinder conservation goals. Our assertion was exceedingly controversial—one preeminent ecologist (in a manuscript review) labeled it “pure rubbish.”

This example is not used to bemoan our experience regarding this particular issue nor to comment on the state of biodiversity research. It is a heuristic for why we need to be cognizant of philosophical concepts, such as theory dependence, when addressing conservation issues that ecologists are being called upon to address. The case studies of Kareiva et al. (2018) illustrate that our experience is far from rare for those working in conservation fields. We suggest, as do Kareiva et al. (2018), that a philosophical reflection of our core ecological tenets is necessary to meet the environmental challenges facing society.

## 7 MOVING FORWARD

The first step in recognizing the relevance of concepts such as normal science and theory dependence is *knowing that there are such concepts*. Simple awareness of such philosophical issues is the first step toward integrating them into ecological discourse. Students (and post‐docs, faculty, and any other practicing ecologist) often become interested in such ideas when they are made aware of them. Philosophically oriented discussions require us to be specific about our semantics and precise definitions are needed to adequately advance scientific exploration. Many unnecessary arguments or disagreements can be resolved by clarifying what it is we are talking about (with normal science the obvious example herein). Furthermore, discussions on philosophies of science inevitably influence our metacognition—the knowledge of and ability to regulate one's thinking. Basic philosophy of ecology training should be standard for students. If past experiences determine how we conduct science, then diverse perspectives may yield more insightful ways to design, conduct, and interpret research. Students should be encouraged to gain experience in multiple scientific subdisciplines (and nonscientific fields) to expand their potential as scientists. Extending this further, ways of viewing nature are deeply embedded in the practices and norms of different cultures, and recognizing these views may serve as a bridge among those with diverse backgrounds having different worldviews (e.g., Reid et al., 2021).

From a practical standpoint, the concepts discussed in this paper are useful for the way the science enterprise operates, for example, regarding grant funding allocation. This can be couched in Kuhn's (1959) essential tension perspective. On the one hand, radical hypotheses may be viewed askance by proposal reviewers. If we largely know what the world is like, the process of normal science is to simply articulate and refine existing theories. Alternatives to mainstream ecological or conservation science truisms may lead to fierce opposition (Kareiva et al., 2018), preventing funding of alternative research directions. Some government agencies and nongovernmental organizations may be invested in, economically or politically, certain lines of inquiry or world views (i.e., theory dependence).

On the other hand, the pursuit of transformative research is a target of many funding agencies (Gravem et al., 2017)—transformative implies the need for research that may relate to paradigm shifts or revolutions in a Kuhnian sense. As an illustration, Gravem et al. (2017) offer the following: “Sarah Gravem had suggested that a planned experiment was insufficiently ‘transformative’. Bruce Menge retorted, ‘Do you think Bob Paine knew he was being transformative when he started ripping sea stars off rocks? No! We won't know if something is important until we test it.’” (Note, for context, that Menge was Paine's student.) This sentiment reflects the tension between creativity and constraint in normal science. There is no singular solution to these divergent pressures in a funding context. Yet, effective funding agencies are likely those that have explicit philosophical underpinnings for how they choose target issues/topics and how they allocate funding.

Kuhn's ideas on theory dependence also illuminate how society influences the questions we ask and how we answer them. This calls for a reflection on “normative” statements—those that relate to subjective assessments of what we value, what is “good” or “bad,” and how we think the world *should* be. We suggest that addressing any ecological challenge should start with a delineation of which components are factual and which are normative (we acknowledge this is often a blurry, albeit important line). The statement that a species is extinct is based on factual evidence; a statement of whether the extinction is good or bad is normative. Kuhn's ideas of theory dependence relate to both types of statements, as our training and personal experiences can affect both what we view as factual and how we think the world should be. Ecologists may strive to reduce bias in the practice of science by minimizing outside influences on fact‐finding—a task that can be especially difficult in applied ecology contexts. But practicing science neither protects us from outside influences nor absolves us of the responsibility to consider them. The expression “follow the science” can be interpreted in many ways, some of which are based on personal or political viewpoints. As such, the contemporary refrain in public discourse to “follow the science” as *the* solution to problems is oversimplistic and can be misleading.

This essay repeats what other ecologists have called for—a more direct integration of philosophical underpinnings into our field. As with other scientific fields, ecology is historical, with its history molding the way ecologists practice science now and into the future. With many pressing environmental challenges, the world relies on ecologists to provide insight and guidance. As such, ecology is not isolated from the societal context in which it is embedded. We are less likely to effectively address challenges without a grounded and informed philosophy of what we are doing and why/how we are doing it. Kuhn's writings, on topics such as normal science and theory dependence, are foundations for working toward a better understanding of the natural world and our place in it.

## AUTHOR CONTRIBUTIONS

**Craig A. Layman:** Conceptualization (lead); investigation (lead); writing – original draft (lead); writing – review and editing (equal). **Andrew L. Rypel:** Funding acquisition (lead); investigation (supporting); writing – review and editing (equal).

## FUNDING INFORMATION

ALR was supported by the Peter B. Moyle and California Trout Endowment for Coldwater Fish Conservation. Funding was also provided by the California Agricultural Experimental Station of the University of California Davis, grant number CA‐D‐WFB‐2467‐H.

## CONFLICT OF INTEREST STATEMENT

We declare no conflict of interest.

## PERMISSION TO REPRODUCE

Not applicable.

## Data Availability

Not applicable.
